# Automated Segmentation and Quantification of White Matter Hyperintensities in Acute Ischemic Stroke Patients with Cerebral Infarction

**DOI:** 10.1371/journal.pone.0104011

**Published:** 2014-08-15

**Authors:** Jang-Zern Tsai, Syu-Jyun Peng, Yu-Wei Chen, Kuo-Wei Wang, Chen-Hua Li, Jing-Yi Wang, Chi-Jen Chen, Huey-Juan Lin, Eric Edward Smith, Hsiao-Kuang Wu, Sheng-Feng Sung, Poh-Shiow Yeh, Yue-Loong Hsin

**Affiliations:** 1 Department of Electrical Engineering, National Central University, Jhongli City, Taoyuan County, Taiwan; 2 Department of Computer Science and Information Engineering, National Central University, Jhongli City, Taoyuan County, Taiwan; 3 Department of Neurology, Landseed Hospital, Pingzhen City, Taoyuan County, Taiwan; 4 Department of Neurology, National Taiwan University Hospital, Taipei City, Taiwan; 5 Department of Medical Imaging, Landseed Hospital, Pingzhen City, Taoyuan County, Taiwan; 6 Department of Radiology, Taipei Medical University-Shuang Ho Hospital, New Taipei City, Taiwan; 7 Department of Neurology, Chi-Mei Medical Center, Tainan City, Taiwan; 8 Department of Clinical Neurosciences, Radiology and Community Health Sciences, University of Calgary, Calgary, Alberta, Canada; 9 Division of Neurology, Department of Internal Medicine, Ditmanson Medical Foundation Chia-Yi Christian Hospital, Chiayi City, Taiwan; 10 Department of Neurology, Chung Shan Medical University and Chung Shan Medical University Hospital, Taichung City, Taiwan; 11 Biomedical Electronics Translational Research Center, National Chiao Tung University, Hsinchu City, Taiwan; University of Münster, Germany

## Abstract

White matter hyperintensities (WMHs) of presumed vascular origin are common in ageing population, especially in patients with acute cerebral infarction and the volume has been reported to be associated with mental impairment and the risk of hemorrhage from antithrombotic agents. WMHs delineation can be computerized to minimize human bias. However, the presence of cerebral infarcts greatly degrades the accuracy of WMHs detection and thus limits the application of computerized delineation to patients with acute cerebral infarction. We propose a computer-assisted segmentation method to depict WMHs in the presence of cerebral infarcts in combined T1-weighted, fluid attenuation inversion recovery, and diffusion-weighted magnetic resonance imaging (MRI). The proposed method detects WMHs by empirical threshold and atlas information, with subtraction of white matter voxels affected by acute infarction. The method was derived using MRI from 25 hemispheres with WMHs only and 13 hemispheres with both WMHs and cerebral infarcts. Similarity index (SI) and correlation were utilized to assess the agreement between the new automated method and a gold standard visually guided semi-automated method done by an expert rater. The proposed WMHs segmentation approach produced average SI, sensitivity and specificity of 83.142±11.742, 84.154±16.086 and 99.988±0.029% with WMHs only and of 68.826±14.036, 74.381±18.473 and 99.956±0.054% with both WMHs and cerebral infarcts in the derivation cohort. The performance of the proposed method with an external validation cohort was also highly consistent with that of the experienced rater.

## Introduction

White matter hyperintensities (WMHs), visible in periventricular and subcortical white matter in T2-weighted magnetic resonance imaging (MRI), could be a radiological manifestation in several intracranial diseases, including multiple sclerosis [1], dementia [2], large [3] and small vascular diseases of hypertensive vasculopathy and cerebral amyloid angiopathy [4]–[7]. WMHs of presumed vascular origin is one of the neuroimaging features of cerebral small vessels disease, including small subcortical infarcts, lacunes, perivascular spaces microbleeds and brain atrophy [8]. WMHs are common in patients with acute cerebral infarction [8] and its presence increases the risk of stroke, cognitive impairment and death [9]. More severe events of cerebral ischemia were associated with WMHs [10]. The volumes of WMHs are associated with cognitive impairment in lobar intracerebral hemorrhage (ICH) [5] and the progression of WMHs is associated with cognitive impairment [11] and incident ICH in follow-up [12]. Higher burden of WMHs is associated with worse outcomes after ischemic stroke [13].

The severity of WMHs could be accessed by several methods, from the visual scoring systems [11], [14]–[15] or semi-automated [12] to automated methods of analysis [16]–[20]. A semi-automated method was reported to be better correlated with WMHs progression than visual scoring systems [21]. However, semi-automated methods requiring human inputs are subjective, time-consuming, laborious, error prone, and vulnerable to intra- and inter-rater variability. An automated method is desirable to provide efficient, reproducible, and reliable WMHs segmentation and volume quantization.

Few automated WMHs quantification techniques have been reported in the literature for patients with acute cerebral infarction. Shi et al. proposed an automated computational method for quantification of the WMHs in the T1-weighted (T1w), fluid attenuation inversion recovery (FLAIR) and diffusion-weighted imaging (DWI) with the presence of acute cerebral infarcts based on mathematical morphological operations [22].

In this study, we emphasize how to avoid the deterioration caused the presence of acute cerebral infarcts in the development of the proposed WMHs segmentation method. This method detects WMHs and cerebral infarcts by their histographic characteristics in combined T1w, FLAIR, and DWI sequences. The proposed method is further strengthened by detecting and eliminating spurious WMHs arising from postischemic edema of the infarct as well as blurred intensity of the gray/white matter junction. The performance of this automated segmentation approach was verified with an external validation cohort with respect to visually guided semi-automated segmentation by an experienced neurologist.

## Materials and Methods

### Subjects and MR imaging protocol

Thirty patients with acute ischemic stroke admitted to Landseed Hospital, a participating teaching hospital in Taiwan Stroke Registry [23], during January–June, 2011, were recruited in this study. A written informed consent was obtained from each participating patient. The protocol of this research had been approved and monitored by Landseed Hospital Institutional Review Board. The average interval between reported stroke onset and MR scanning was 82.19±33.35 hours (mean ± standard deviation) in the derivation cohort. The MRI showed that all the 30 patients had WMHs.

The 30 patients recruited were divided into a derivation cohort (20 patients, 8 females and 12 males, 69.9±12.2 years old) and a validation cohort (10 patients, 3 females and 7 males, 63.4±11.9 years old). In the derivation cohort, one of the 40 hemispheres was excluded because it only contained old large infarct without acute ischemic stroke; another was excluded due to massive cerebral infarcts on the whole hemisphere to completely mask the WMHs. We finally had 25 hemispheres with WMHs only and 13 hemispheres with both WMHs and cerebral infarcts. In the validation cohort, among the 20 hemispheres, 13 had WMHs only and the other 7 had both WMHs and cerebral infarcts.

All MRI data of the patients were collected using a Signa HDxt 1.5T (GE healthcare Milwaukee, WI) scanner with an eight-channel phased-array neurovascular coil to obtain the T1w, FLAIR, and DWI sequences. The parameters for acquiring T1w sequence were repetition time (TR) = 2400 ms, echo time (TE) = 24 ms, echo train length (ETL) = 6, field of view (FOV) = 230 mm, number of excitations (NEX) = 2, matrix size = 288×192, slice thickness = 5 mm, and slice gap = 1 mm. The parameters for acquiring FLAIR sequence were TR = 8000 ms, TE = 151 ms, ETL = 36, FOV = 230 mm, NEX = 1.5, matrix size = 288×288, slice thickness = 5 mm, and slice gap = 1 mm. The parameters for acquiring DWI sequence were TR = 6000 ms, TE = 82.8 ms, ETL = 1, FOV = 230 mm, NEX = 2, matrix size = 128×128, slice thickness = 5 mm, and slice gap = 1 mm.

### The semi-automated segmentation of WMHs

The neurologist rater (Y.W. Chen) did the semi-automated WMHs segmentation based on the protocol developed by Stroke Service, Massachusetts General Hospital. The details of the procedure were previously described in the articles of related research [12], [24]–[25]. In brief, the areas and volumes were determined after the region of interest was created by combination of appropriate determination of signal intensity threshold and manual editing. The location of WMHs and acute infarct was recorded based on the corresponding neuroanatomy in the imaging.

### Histographic characterization of WMHs

The histograms of the semi-automatically demarcated WMHs were studied. Specifically, the lowest bounds of the normalized intensity of these semi-automatically demarcated WMHs in the 38 available hemispheres of the derivation cohort, were analyzed.

### The automated WMHs segmentation procedure

**Figure 1:**
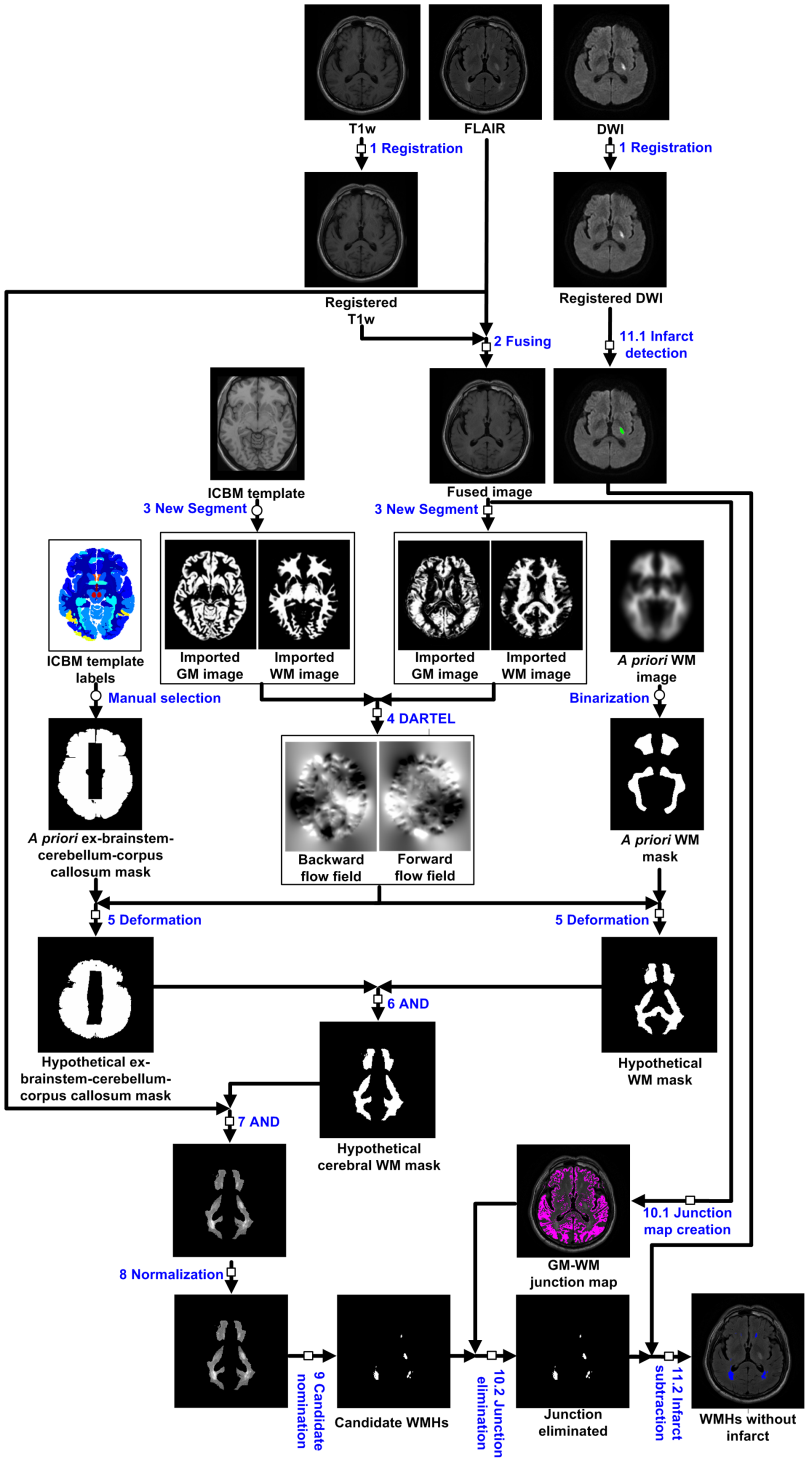
The flow diagram of the automated WMHs segmentation algorithm. Each represents a step that must be performed for each individual subject; each represents a step that needs to be performed only once for all subjects.

As depicted in Fig. 1, the automated WMHs segmentation of a single hemisphere consisted of 11 steps as described below.

#### Step 1

Registration. To correct for differences due to subject head movement, the T1w image and DWI were registered to the FLAIR image by a rigid registration based on normalized mutual information [26].

#### Step 2

**Figure 2:**
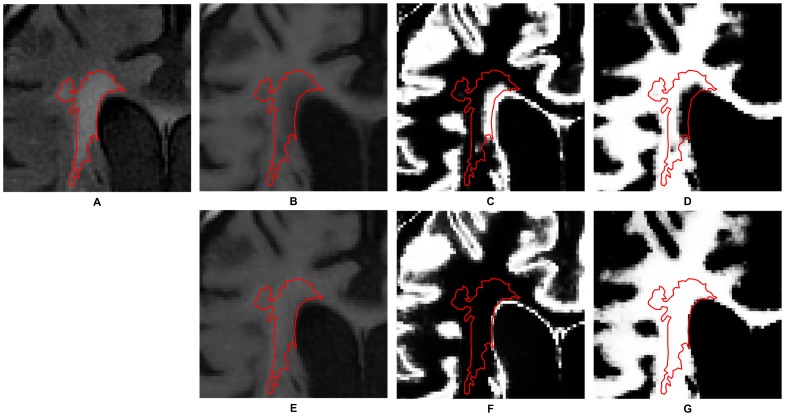
The effect of fusing T1w and FLAIR images. The red contour in these images represents the position of WMHs delineated semi-automatically by the experienced neurologist. (A) An FLAIR image. (B) The registered T1w image. (C) The probabilistic map representing the gray matter segmented from B. (D) The probabilistic map representing the white matter segmented from B. (E) The registered T1w image after being fused with the FLAIR image. (F) The probabilistic map representing the gray matter segmented from E. (G) The probabilistic map representing the white matter segmented from E. Note that in the probability maps, higher intensity corresponds to higher probability.

Fusing. The registered T1w image was fused with the FLAIR image to avoid incorrect segmentation due to the similarity between the intensities of the WMHs and the gray matter. The fusing formula was *V*_fusion_ = *kV*_registered T1w_+(1−*k*)*V*_FLAIR_, where *k* was the weighting coefficient that was set at 0.8 [22], and *V*_fusion_, *V*_registered T1w_ and *V*_FLAIR_ represent the fused image, registered T1w image and FLAIR image, respectively. The effect of fusing T1w and FLAIR images is showed in Fig. 2.

#### Step 3

The New Segment module of Statistical Parametric Mapping 8 (SPM8, Wellcome Department of Cognitive Neurology, London, UK, http://www.fil.ion.ucl.ac.uk/spm/) was applied on the fused T1w image to create probabilistic maps of gray and white matter, i.e., the imported gray matter image and the imported white matter image [27].

#### Step 4

The imported white matter image and the imported gray matter image were utilized to create the transformation parameters through the DARTEL (Diffeomorphic Anatomical Registration Through Exponential Lie Algebra) module embedded in SPM8 [28].

#### Step 5

Deformation. Deform the *a priori* white matter mask and the *a priori* ex-brainstem–cerebellum–corpus callosum mask into the hypothetical white matter mask and the hypothetical ex-brainstem–cerebellum–corpus callosum mask, respectively, based on the forward flow field and the backward flow field. The *a priori* white matter mask had been obtained from the *a priori* white matter probabilistic map through a binarization operation that kept voxels with probabilities over 0.5. The hypothetical ex-brainstem–cerebellum–corpus callosum mask had been obtained by manually selecting from ICBM (International Consortium for Brain Mapping) template labels (http://www.loni.usc.edu/ICBM/).

#### Step 6

AND. Obtain the hypothetical cerebral white matter mask by an AND operation of the hypothetical ex-brainstem–cerebellum–corpus callosum mask and the hypothetical white matter mask.

#### Step 7

AND. Obtain the FLAIR image within the scope of the hypothetical cerebral white matter mask by an AND operation.

#### Step 8

Normalization. The FLAIR image within the hypothetical cerebral white matter mask was normalized so that its intensity distributed in a standardized range [0, 100].

#### Step 9

Nomination of candidate WMHs. Voxels with normalized intensity higher than a designated threshold, namely 65, were selected as belonging to the candidate WMHs. This threshold level was determined based on histographic characterization of the semi-automatically demarcated WMHs and it, however, can be adjusted manually.

#### Step 10.1

Junction map creation. The fused image was binarized to get a gray/white matter junction map [29], [30]. The rule of the binarization was to assign logic 1 to voxels with intensity between *I*_average, GM_+0.5 *I*_stdev, GM_ and *I*_average, WM_−0.5 *I*_stdev, WM_, where *I*_average, GM_ and *I*_stdev, GM_ represent the average value and the standard deviation, respectively, of the voxel intensity of the gray matter and *I*_average, WM_ and *I*_stdev, WM_ represent those of the white matter.

#### Step 10.2

Junction elimination. In the resultant candidate WMHs of Step 9, if a voxel or any of its 8 neighboring voxels was a junction voxel, this voxel was said to be junction-connected. If more than 80% of the voxels in a contiguous region of a candidate WMHs were junction-connected, this candidate WMHs was said to be junction-connected and would be disqualified as a WMHs.

#### Step 11.1

**Figure 3:**
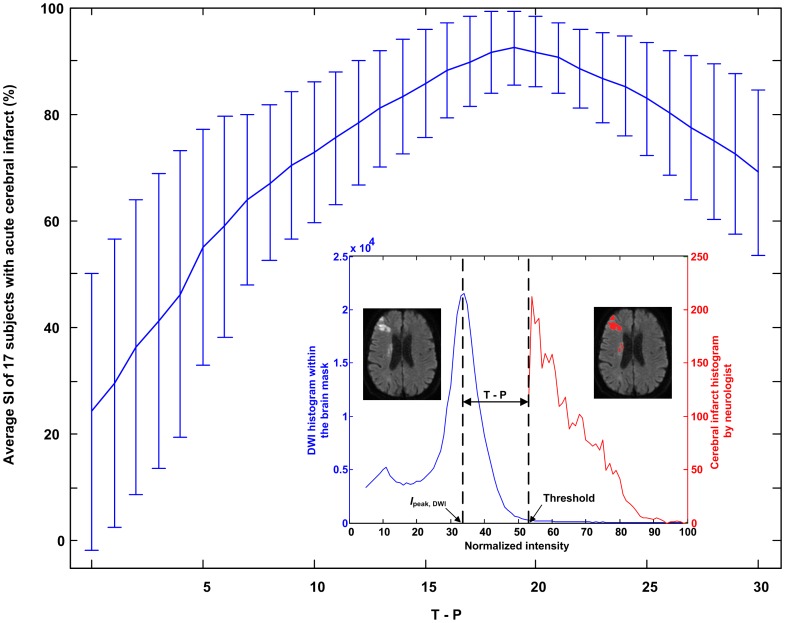
Determination of an optimal threshold for cerebral infarcts segmentation. A preliminary experiment was conducted on 17 subjects with acute cerebral infarction. Shown here are the average SI and standard deviation versus threshold *I*_peak,DWI_+*n*, *n* ranging from 0 to 30. A suitable threshold was *I*_peak,DWI_+19 and the resultant average SI was 92.352±6.944%.

Cerebral infarcts detection. The cerebral infarcts manifested themselves as hyperintensive regions in the registered DWI and could be detected. The brain mask was extracted from the registered whole-brain DWI by using Brain Extraction Tool, a software package developed at FMRIB Centre, University of Oxford, Oxford, United Kingdom (http://www.fmrib.ox.ac.uk/) [31]. The peak of the normalized DWI histogram in a standardized intensity range [0, 100] within the brain mask was identified and denoted by *I*_peak,DWI_. A segmentation of cerebral infarct lesions was conducted using *I*_peak,DWI_+19 as the threshold level, as depicted in Fig. 3 [32].

#### Step 11.2

Cerebral infarcts subtraction. In the resultant WMHs of Step 10.2, the portion that overlapped with the detected cerebral infarcts would be subtracted according to the following rule: All the voxels of any one among the WMHs labels in the FLAIR image would be subtracted if at least 80% of the label's corresponding DWI voxels belonged to an cerebral infarct label; any voxel of other FLAIR WMHs labels would be subtracted if it corresponded to an cerebral infarct voxel; otherwise, it would be kept in the resultant WMHs.

### Quantitative evaluations

The similarity index (SI) was used to evaluate the agreement between the automated and semi-automated segmentation in 25 hemispheres with WMHs only and 13 hemispheres with both WMHs and cerebral infarcts in the derivation cohort. The sensitivity, specificity, and SI [33] were calculated. The Bland–Altman plot evaluated the systematic dissimilarity [34]. The volume measurements agreement evaluation of the proposed algorithm was carried out by calculating the intraclass correlation coefficient (ICC) [35]. The same parameters were further examined in the validation cohort.

## Results

**Figure 4:**
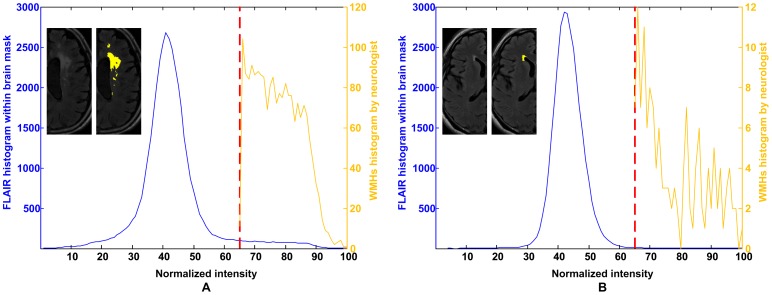
Histographic characterization of WMHs. The intensity histogram of the whole-brain normalized FLAIR image in (A) the right hemisphere of patient #1, of which the semi-automatically demarcated WMHs volume was larger (7.954 ml), and (B) the left hemisphere of patient #20, of which the semi-automatically demarcated WMHs volume was smaller (0.736 ml). The demarcated WMHs in a slice is painted in yellow and shown in the inlet. The lowest bounds of the normalized intensity of the semi-automatically demarcated WMHs were both 65 in the hypothetical cerebral WM mask.

In the histographic characterization of WMHs in the derivation cohort, 38 hemispheres were studied. As an example, the blue curve in Fig. 4(A) is the intensity histogram of the white matter in the hypothetical cerebral white matter mask in the normalized FLAIR image of the right hemisphere of patient #1. The yellow curve is the intensity histogram of the semi-automatically demarcated WMHs, which was 7.954 ml in volume. Similarly, Fig. 4(B) shows the intensity histograms of the left hemisphere of patient #20, of which the semi-automatically demarcated WMHs volume was 0.736 ml. The lowest bounds of the normalized intensity of the semi-automatically demarcated WMHs of the 38 hemispheres ranged from 55 to 75 and averaged 65. Based on this result, the threshold for nominating candidate WMHs was set as 65 in Step 9 of the proposed method.

**Figure 5:**
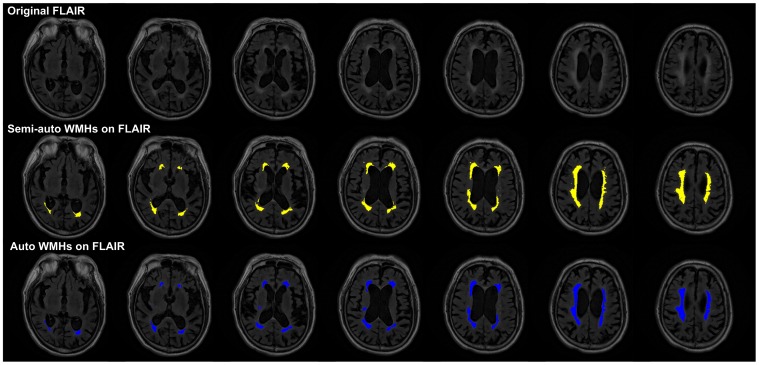
Typical examples of WMHs segmentation. Seven successive axial slices taken from patient #2are shown (upper row: FLAIR image; middle row: yellow regions being the WMHs demarcated by the neurologist; lower row: blue regions being the WMHs demarcated by the proposed algorithm).

**Figure 6:**
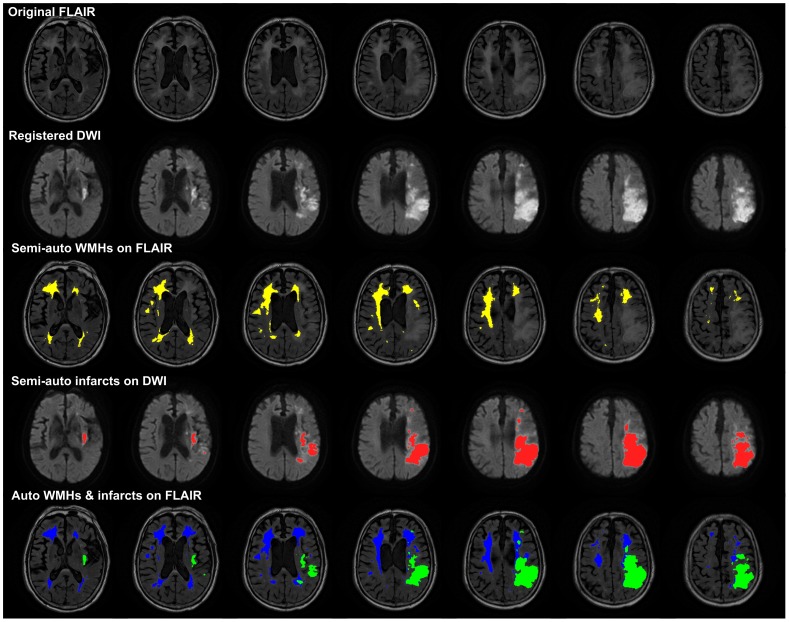
Typical examples of WMHs segmentation with both WMHs and cerebral infarcts. The original FLAIR image, registered DWI, semi-automated WMHs, semi-automated cerebral infarcts, and automated WMHs and cerebral infarcts (top to bottom) were illustrated using patient #17 as a typical example with both WMHs and cerebral infarcts. This figure shows 7 continuous slices of the 23 slices in the whole-brain. It was difficult for the neurologist to recognize the boundary between WMHs and cerebral infarct if they had close contact to each other.

**Figure 7:**
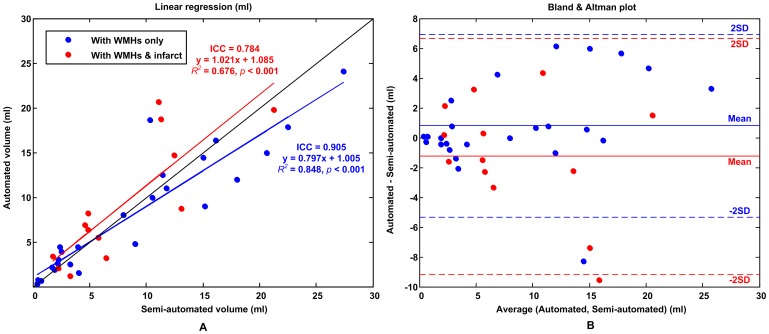
Evaluation of WMHs segmentation results. (A) Linear regression (B) Bland & Altman plot. The blue lines and dots are for the hemisphere with WMHs only; the red lines and dots are for the hemisphere with both WMHs and cerebral infarcts.

The accuracy of the WMHs segmentation algorithm has been evaluated on the 20 patients in the derivation cohort, including 25 hemispheres with WMHs only and 13 hemispheres with both WMHs and cerebral infarcts. The ICC of WMHs delineation was 0.905 for the hemisphere group with WMHs only and 0.784 for the hemisphere group with both WMHs and cerebral infarcts. Fig. 5 and 6, respectively, shows a typical example of automated segmentation for patients with WMHs only and with both WMHs and cerebral infarction. In the image presentation in this paper, the semi-automatically demarcated WMHs and cerebral infarcts by the neurologist are painted yellow and red, respectively; the automatically demarcated WMHs and cerebral infarcts are painted blue and green, respectively. Linear regression analyses resulted in a regression coefficient value of *R^2^* = 0.848 and a regression slope of 0.797 for the hemisphere group with WMHs only, whereas *R^2^* = 0.676 and slope of 1.021 for the hemisphere group with both WMHs and cerebral infarcts, as shown in Fig. 7(A). Bland and Altman plot in Fig. 7(B) shows a slight overestimation (bias of 0.799 ml) for the hemisphere group with WMHs only, and a larger underestimation (bias of −1.248 ml) for the hemisphere group with both WMHs and cerebral infarcts.

We used the SI to indicate the degree of agreement between the WMHs demarcated by the semi-automated and the proposed segmentation methods. It is considered to have high agreement if SI is higher than 0.7 [36], [37]. The SI, sensitivity, and specificity of the WMHs segmentation of each hemisphere in the derivation and validation cohorts were calculated and are shown in Tables 1 and 2, respectively. The average performance of the WMHs segmentation of hemispheres with WMHs only and that of hemispheres with both WMHs and infarcts in the derivation and validation cohorts are also shown in Tables 1 and 2, respectively. The latter has lower SI because of unclear boundary definition due to the coexistence of WMHs and cerebral infarcts. Verified with the semi-automated WMHs delineation as the gold standard, the proposed method has higher SI, sensitivity, and specificity for hemisphere with WMHs only; the SI was (83.142±11.742)% (mean ± standard deviation), the sensitivity was (84.154±16.086)%, and the specificity was (99.988±0.029)% in the derivation cohort. For the hemisphere with both WMHs and cerebral infarct, the SI was (68.826±14.036)%, the sensitivity was (74.381±18.473)%, and the specificity was (99.956±0.054)% in the derivation cohort. For the entire derivation cohort, the SI was (78.244±14.167)%, the sensitivity was (80.811±17.338)%, and the specificity was (99.977±0.042)%.

**Table 1:** Performance of the computer-assisted WMHs segmentation method with the derivation cohort.

Patient	Side	WMHs volume (ml)		Δ Vol.	Demar.	SI	Sen.	Spe.
		Semi-auto.	Auto.					
1	L	Old infarct lesions						
	R	7.954	7.994	0.5%	0.65	96.759%	97.005%	99.996%
2	L	15.019	14.447	−3.8%	0.64	95.266%	93.454%	99.994%
	R	11.459	12.490	9.0%	0.66	94.843%	99.112%	99.984%
3	L	22.525	17.862	−20.7%	0.62	87.954%	78.849%	99.998%
	R	Massive infarcts						
4	L	15.159	9.018	−40.5%	0.59	73.798%	58.850%	99.999%
	R*	13.072	8.747	−33.1%	0.60	80.089%	66.840%	100.000%
5	L	3.945	4.405	11.7%	0.66	91.489%	96.827%	99.990%
	R*	5.766	5.495	−4.7%	0.66	79.168%	77.311%	99.983%
6	L*	11.335	18.739	65.3%	0.73	59.643%	79.124%	99.853%
	R	10.338	18.624	80.2%	0.75	61.475%	86.112%	99.854%
7	L*	12.495	14.728	17.9%	0.56	51.877%	56.512%	99.899%
	R	20.637	14.965	−27.5%	0.58	83.989%	72.448%	100.000%
8	L	9.009	4.746	−47.3%	0.57	68.428%	52.238%	99.999%
	R*	6.452	3.208	−50.3%	0.56	66.422%	49.725%	100.000%
9	L	18.012	12.016	−33.3%	0.60	79.194%	66.012%	99.998%
	R	11.779	11.008	−6.5%	0.65	92.412%	89.391%	99.993%
10	L	2.362	4.427	87.4%	0.74	64.883%	93.246%	99.967%
	R*	4.839	8.167	68.8%	0.74	55.128%	74.084%	99.931%
11	L	4.041	1.533	−62.1%	0.55	55.008%	37.939%	100.000%
	R*	3.297	1.143	−65.3%	0.58	51.497%	34.677%	100.000%
12	L	10.573	9.938	−6.0%	0.65	92.420%	89.647%	99.993%
	R*	4.625	6.901	49.2%	0.68	61.933%	77.173%	99.952%
13	L*	4.841	6.344	31.0%	0.67	84.677%	97.821%	99.980%
	R*	1.767	3.391	91.9%	0.73	62.781%	91.642%	99.978%
14	L	1.692	2.148	27.0%	0.68	77.813%	88.305%	99.989%
	R	0.412	0.339	−17.6%	0.65	79.570%	72.549%	99.999%
15	L	1.892	1.897	0.2%	0.65	94.503%	94.614%	99.998%
	R	2.548	3.944	54.8%	0.70	77.816%	99.130%	99.979%
16	L*	21.281	19.794	−7.0%	0.64	96.309%	92.945%	100.000%
	R	16.142	16.341	1.2%	0.66	94.468%	95.050%	99.987%
17	L	27.383	24.070	−12.1%	0.63	93.486%	87.832%	100.000%
	R*	11.125	20.656	85.7%	0.65	64.066%	91.511%	99.856%
18	L	2.233	3.057	36.9%	0.68	80.737%	95.635%	99.986%
	R*	2.273	2.074	−8.8%	0.66	81.142%	77.583%	99.995%
19	L	3.259	2.499	−23.3%	0.61	86.796%	76.672%	100.000%
	R	2.184	2.567	17.5%	0.68	86.646%	94.235%	99.993%
20	L	0.736	0.683	−7.2%	0.65	94.198%	90.789%	100.000%
	R	0.465	0.756	62.5%	0.70	74.603%	97.917%	99.996%
Total (n = 38)			Mean	8.5%	0.65	78.244%	80.811%	99.977%
			STDEV	43.1%	0.05	14.167%	17.338%	0.042%
Hemispheres with WMHs only (n = 25)			Mean	3.2%	0.65	83.142%	84.154%	99.988%
			STDEV	37.9%	0.05	11.742%	16.086%	0.029%
Hemispheres with both WMHs and infarcts (n = 13)			Mean	18.5%	0.65	68.826%	74.381%	99.956%
			STDEV	51.7%	0.06	14.036%	18.473%	0.054%

Note: Δ Vol. = (Auto. vol. – Semi-auto. vol.)/Semi-auto. vol.; Demar. = the lowest intensity of the WMHs demarcated by the neurologist; SI = similarity index; Sen. = sensitivity; Spe. = specificity; Stdev = standard deviation; L (or R) = left (or right) hemisphere containing WMHs only; L* (or R*) = left (or right) hemisphere containing both WMHs and cerebral infarct lesions.

**Table 2:** Performance of the computer-assisted WMHs segmentation method with the validation cohort.

Patient	Side	WMHs volume (ml)		Δ Vol.	SI	Sen.	Spe.
		Semi-auto.	Auto.				
1	L	12.421	12.500	0.6%	92.212%	92.508%	99.985%
	R	11.605	13.954	20.2%	87.621%	96.487%	99.959%
2	L	3.408	4.373	28.3%	84.484%	96.445%	99.983%
	R*	3.210	5.624	75.2%	71.207%	97.987%	99.961%
3	L*	0.864	0.758	−12.3%	79.235%	74.359%	99.998%
	R	0.944	1.143	21.1%	86.624%	95.775%	99.997%
4	L*	4.059	2.736	−32.6%	80.542%	67.422%	100.000%
	R	5.531	4.741	−14.3%	89.863%	83.450%	99.998%
5	L	1.809	1.814	0.3%	88.501%	88.630%	99.997%
	R	1.376	1.329	−3.4%	92.008%	90.421%	99.999%
6	L	1.681	1.065	−36.6%	77.601%	63.401%	100.000%
	R*	1.095	1.259	15.0%	80.247%	86.283%	99.996%
7	L	1.413	1.825	29.2%	85.287%	97.714%	99.993%
	R*	0.812	0.864	6.4%	71.807%	74.129%	99.996%
8	L	1.208	0.823	−31.9%	81.039%	68.122%	100.000%
	R	1.065	0.791	−25.7%	80.682%	70.297%	99.999%
9	L*	0.664	0.630	−5.2%	89.888%	87.591%	99.999%
	R	0.223	0.184	−17.5%	59.524%	54.348%	99.999%
10	L	1.001	0.630	−37.1%	76.238%	62.097%	100.000%
	R*	0.929	0.420	−54.8%	62.275%	45.217%	100.000%
Total (n = 20)			Mean	−3.8%	80.844%	79.634%	99.993%
			STDEV	30.2%	9.167%	15.891%	0.012%
Hemispheres with WMHs only (n = 13)			Mean	−5.1%	83.206%	81.515%	99.993%
			STDEV	24.3%	8.756%	15.588%	0.012%
Hemispheres with both WMHs and infarcts (n = 7)			Mean	−1.2%	76.457%	76.141%	99.993%
			STDEV	41.1%	8.845%	17.082%	0.014%

Note: Δ Vol. = (Auto. vol. – Semi-auto. vol.)/Semi-auto. vol.; SI = similarity index; Sen. = sensitivity; Spe. = specificity; Stdev = standard deviation; L (or R) = left (or right) hemisphere containing WMHs only; L* (or R*) = left (or right) hemisphere containing both WMHs and cerebral infarct lesions.

The performance of our algorithm was further evaluated with the validation cohort. For the hemispheres with WMHs only, the SI was (83.206±8.756)% (mean ± standard deviation), the sensitivity (81.515±15.588)%, and the specificity (99.993±0.012)%. For the hemispheres with both WMHs and cerebral infarcts, the SI was (76.457±8.845)%, the sensitivity (76.141±17.082)%, and the specificity (99.993±0.014)%. For the entire validation cohort, the SI was (80.844±9.167)%, the sensitivity (79.634±15.891)%, and the specificity (99.993±0.012)%.

The proposed automated WMHs segmentation method was developed on a personal computer with Intel Core i5 CPU, 2.67 GHz processor speed, and 4GB RAM. The WMHs segmentation procedure was carried out mainly with a MATLAB program (The MathWorks, Inc., Natick, MA). It took our personal computer less than 90 seconds to execute from Step 1 through Step 11, excluding Step 4. Involving the creation of the transformation parameters with DARTEL module program, Step 4 took nearly 15 minutes.

## Discussion

Automated segmentation of WMHs has been reported by many research teams [16]–[20], [22], [38]–[46]. However, our literature survey found that, so far, Shi et al. [22] are the only team that has considered the presence of cerebral infarcts in addition to WMHs. Our proposed WMHs segmentation method also emphasizes coexistence of acute cerebral infarcts, the associated surrounding edema and WMHs; moreover, our method can eliminate spurious WMHs in gray/white matter junction.

Separation of gray and white matter of the registered T1w in Step 3 could be degraded by WMHs, which had lower intensities than the surrounding white matter and could be wrongly categorized into gray matter. As shown in Fig. 2 (C) and (D), the segmentation of gray and white matter, respectively, from the registered T1w image Fig. 2 (B) was not satisfactory so that the segmented gray matter overran the WMHs region delineated by the neurologist rater. The effect of the fusing operation in Step 2 was to offset the intensity of the WMHs so that they would not be wrongly categorized. In Fig. 2 (F) and (G), it is evident that the gray and white matter segmented from the fused image Fig. 2 (E) were much more consistent with the WMHs delineated by the neurologist rater.

The consistency between the automated and the semi-automated lesions segmentation on the hemispheres with WMHs only indicates a high reliability of the method in the absence of cerebral infarct lesions. The linear regression analyses and Bland and Altman plot shown in Fig. 7 indicate an excellent volume and spatial agreement for different lesion sizes of from 0.412 to 27.383 ml. As shown in Table 1, the mean SI value was 83% in the derivation cohort with WMHs only, indicating outstanding voxel agreement between the automated and semi-automated segmentations. The automated and semi-automated segmentation had a lower volume and spatial agreement if the hemispheres had both WMHs and cerebral infarcts. That was due to the rater's variation in WMHs delineation with the influence of cerebral infarcts.

This research focused on supratentorial WMHs. The WMHs in brainstem and cerebellum were excluded and corpus callosum was conventionally not included in the segmentation of WMHs. The proposed algorithm used the hypothetical white matter mask and the hypothetical ex-brainstem–cerebellum–corpus callosum mask to conceal these regions in Step 7. These masks must be personalized to be anatomically accurate. They were obtained by inversely deforming (in Step 5) the *a priori* white matter mask and the *a priori* ex-brainstem–cerebellum–corpus callosum mask to match the individual brain shapes using the SPM plus DARTEL.

In view of interrater variability and subjectiveness, the selection of a proper threshold value in Step 9 is critical to the performance of the proposed method. Since there won't be a universal threshold value to suit all raters in any scanning circumstance, the proposed method allows for manual adjustment of the threshold value. This flexibility also helps attain higher volume and spatial agreement in WMHs segmentation with different MR scanners or protocols.

The proposed method used a constant threshold, namely 65 of the normalized intensity in the FLAIR image, in Step 9 for nominating candidate WMHs. On the contrary, the threshold level used by the neurologist to demarcate the WMHs was not constant, as shown in Table 2.

Biological variance and varying image quality, such as contrast-to-noise ratio (CNR), have been the main causes of low SI. For example, the neurologist's threshold in demarcating the right hemisphere of patient #6, 75 in terms of normalized FLAIR intensity, resulting in the second lowest SI (61.475%) among the 25 hemispheres with WMHs only, despite of good sensitivity of 86.112%. The threshold value 75 used was higher than 65 used for our automated segmentation. This higher threshold may be attributed to a low CNR between the WMHs and surrounding white matter of the FLAIR intensity of patient #6 compared to those of other patients. For another example, the neurologist's threshold in demarcating the left hemisphere of patient #11 was 55 to result in the lowest SI (55.008%) and the lowest sensitivity (37.939%) among the 25 hemispheres with WMHs only. This lower threshold may be attributed to a high CNR between the WMHs and surrounding white matter of the FLAIR intensity of patient #11 compared to those of other patients.

**Figure 8:**
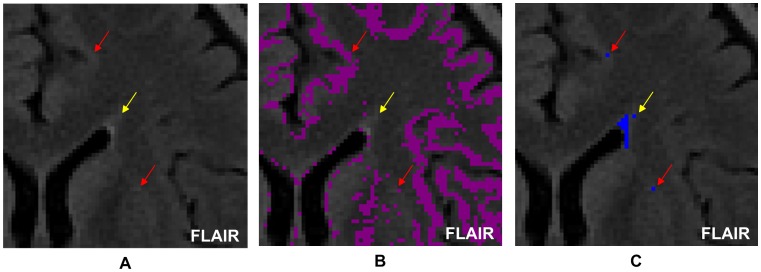
Removal of hyperintensive junction-connected regions from candidate WMHs. (A) The three arrows point to three hyperintensities on a FLAIR image; (B) Shown in purple is the gray/white matter junction created in Step 10.1. This figure shows that the two hyperintensities pointed to by the red arrows were junction-connected, whereas that pointed to by the yellow arrow was not junction-connected; (C) The three hyperintensities were all nominated as candidate WMHs in Step 9. However, in Step 10.2, the two pointed to by the red arrows would be eliminated from the candidate WMHs and only the one pointed to by the yellow arrow would remain in the candidate WMHs.

In the resultant candidate WMHs of Step 9, some were not real WMHs; instead, they were just hyperintensive regions belonging in the gray/white matter junction and should be removed from the candidate WMHs, which would be done in Step 10.2. In Step 10.1, a gray/white matter junction map was created from the fused image to represent the brain regions with blurred gray/white matter transition. In Step 10.2, junction-connected hyperintensities were identified and eliminated. Fig. 8 shows an example of this action.

WMHs and acute cerebral infarcts appear similar in the FLAIR image so that there is no clear borderline between them when they adjoin each other. As a result, the semi-automated segmentation usually fails to produce a reliable demarcation of WMHs if there is a concomitant cerebral infarct region. The automated segmentation is advantageous in that the cerebral infarcts can be demarcated separately from the DWI and be subtracted from the demarcated WMHs from the FLAIR image. However, a complete elimination of cerebral infarcts from demarcated WMHs can be hindered by postischemic peri-infarct edema of acute cerebral infarcts. The edema reduces the peripheral intensity of the cerebral infarcts in the DWI. Hence, the detected size of acute cerebral infarcts in the DWI is usually smaller than its actual size and its corresponding area in the FLAIR. Consequently, directly subtracting the demarcated cerebral infarct of Step 11.1 from the demarcated WMHs of Step 10.2 will lead to spurious WMHs labels.

**Figure 9:**
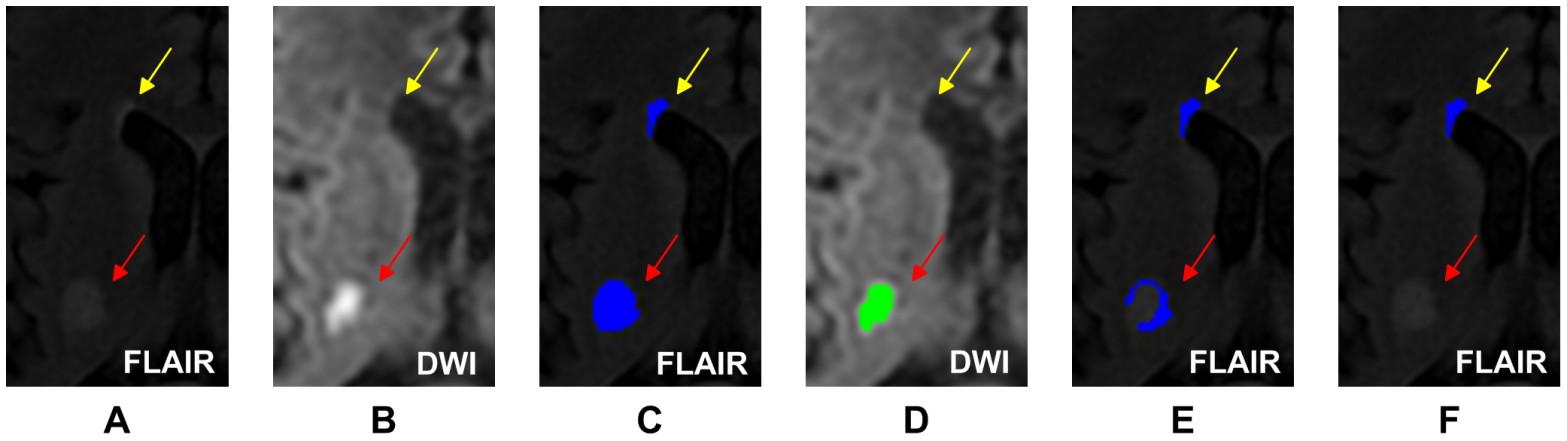
Elimination of a spurious WMHs due to postischemic peri-infarct edema. (A) The hyperintensities pointed to by the yellow and red arrows were WMHs and cerebral infarct, respectively, in FLAIR; (B) the infarct appeared hyperintensive in DWI, with a peripheral intensity reduction due to peri-infarct edema; (C) both regions were detected as WMHs in Step 10.2; (D) the green region pointed to by the red arrow was the infarct detected in Step 11.1; (E) the blue regions were the result of subtracting the result of Step 11.1 from the result of Step 10.2; the blue region pointed to by the red arrow was a spurious WMHs due to peri-infarct edema; (F) the result of Step 11.2; the spurious WMHs due to peri-infarct edema was eliminated successfully.

An example to illustrate how Step 11.2 in our algorithm dealt with this spurious WMHs problem is shown in Fig. 9. In the FLAIR image shown in Fig. 9 (A), the yellow and red arrows point to the areas of WMHs and a cerebral infarct, respectively. As shown in Fig. 9(C), the two were both detected as WMHs in Step 10.2. In the corresponding DWI shown in Fig. 9(B), the cerebral infarct pointed to by the red arrow had high intensity and was detected as a cerebral infarct in Step 11.1, as shown in Fig. 9(D). Note that the detected cerebral infarct, represented by the green region in Fig. 9(D), was smaller than the actual cerebral infarct because its peripheral intensity had been reduced due to postischemic peri-infarct edema. In Fig. 9(F), the region pointed to by the yellow arrow was the detected WMHs. The crescent-shaped region pointed to by the red arrow was actually part of the cerebral infarct. It was generated there because the detected cerebral infarct region in Step 11.1 was smaller than the real infarct size. It was at last successfully eliminated according to the rule established in Step 11.2. Fig. 9(E) represents a hypothetical result of directly subtracting the detected infarct region of Step 11.1 from the detected WMHs regions of Step 10.2 and it does not represent a result of any step in our method.

## Conclusions

In this study, we developed a computer-assisted segmentation method for quantification of WMHs with or without the influence of cerebral infarctions in the T1w, FLAIR, and DWI images. The proposed automated method detects WMHs from a hypothetical cerebral white matter region based on the image intensity histogram. This approach attains high SI and correlation between the automated and semi-automated segmentation of WMHs for the hemispheres with WMHs only. In the presence of cerebral infarcts in addition to WMHs, a reliable algorithm is adopted to demarcate the cerebral infarcts to be subtracted from the WMHs. Thus, the proposed algorithm is suitable for segmentation of WMHs in acute ischemic stroke patients with or without cerebral infarction. Moreover, the threshold for WMHs detection can be adjusted to accommodate to different MRI scanners and sequences and to systemically decrease the variation from the patients, machines, and raters. It could provide both real-time information in the scenario of emergent medicine and a subjective, reliable basis of longitudinal and cross-sectional imaging study.
